# Supplementary material for: The effects of haploid selection on Y chromosome evolution in two closely related dioecious plants
Source: Evol Lett. 2018 Jun 22;2(4):368–77. doi: 10.1002/evl3.60 (PMC6121804; doi:10.1002/evl3.60)
Supplement: Supplementary file 1 — Figure S1. Principle Component Analysis (PCA) of gene expression for 14 different tissue samples in Rumex hastatulus and R. rothschildianus. Figure S2. Fraction of genes in each FPKM expression bin for Rumex hastatulus and R. rothschildianus pollen for hemizygous (black) and XY (grey) genes. Figure S3. Tissue expression bias of different gene groups in Rumex hastatulus and R. rothschildianus. Figure S4. Tissue expression bias of different gene groups in Rumex hastatulus and R. rothschildianus. Table S1. Counts of differentially expressed genes in two tissue comparisons of Rumex hastatulus and R. rothschildianus. Table S2. Counts of XY genes with no allelic bias and Y and X biased allelic expression in Rumex hastatulus and R. rothschildianus. [file EVL3-2-368-s001.docx]

**Supplementary Figure captions**

Supplementary Fig. 1: Principle Component Analysis (PCA) of gene expression for 14 different tissue samples in *Rumex hastatulus* and *R. rothschildianus*.

Supplementary Fig. 2: Fraction of genes in each FPKM expression bin for *Rumex hastatulus* and *R. rothschildianus* pollen for hemizygous (black) and XY (grey) genes. The Median value is represented by a dashed line.

Supplementary Fig. 3. Tissue expression bias of different gene groups in *Rumex hastatulus and R. rothschildianus*. Bar segments represent fraction of genes with significant differential expression bias in two pairwise tissue comparisons. The sex-linked group refers to the combined group of XY and hemizygous genes representing the gene content of the sex chromosome prior to Y degeneration. However, there exists an ascertainment bias in this group as more SNP segregation patterns were used to identify XY linked genes relative to hemizygous genes. The sex linked-corrected group represents the group of genes that remain when this bias is corrected for.

Supplementary Fig. 4: Tissue expression bias of different gene groups in *Rumex hastatulus and R. rothschildianus*. Bar segments represent fraction of genes with significant differential expression bias in two pairwise tissue comparisons. Orthologs of XY genes are compared to other autosomal genes from the same genetic background.

**Supplementary tables**

Supplementary Table 1: Counts of differentially expressed genes in two tissue comparisons of *Rumex hastatulus and R. rothschildianus*.

| *Rumex hastatulus* | | | | |
| --- | --- | --- | --- | --- |
| Comparison of Differential expression | **Direction of overexpression** | **# Overexpressed XY** | **# Overexpressed Autosomal** | **# Overexpressed X-only** |
| Leaf and pollen | Leaf | 738 (60%) | 212 (79%) | 96 (81%) |
|  | Pollen | 486 (40%) | 54 (20%) | 23 (19%) |
| Flower bud and pollen | Flower bud | 389 (54%) | 116 (68%) | 39 (70%) |
|  | Pollen | 335 (46%) | 54 (32%) | 17 (30%) |
| Correcting for degeneration of Y | | | | |
| Comparison of Differential expression | **Direction of overexpression** | **# Overexpressed (XY and X -only)** | **# Overexpressed (XY and X -only + ascertainment bias removed)** | |
| Leaf and pollen | Leaf | 834 (62%) | 299 (55%) | |
|  | Pollen | 509 (38%) | 242 (45%) | |
| Flower bud and pollen | Flower bud | 428 (55%) | 148 (45%) | |
|  | Pollen | 352 (45%) | 182 (55%) | |

| *Rumex rothschildianus* | | | | |
| --- | --- | --- | --- | --- |
| Comparison of Differential expression | **Direction of overexpression** | **# Overexpressed XY** | **# Overexpressed Autosomal** | **# Overexpressed X-only** |
| Leaf and pollen | Leaf | 57 (32%) | 295 (67%) | 136 (80%) |
|  | Pollen | 123 (68%) | 145 (33%) | 35 (20%) |
| Flower bud and pollen | Flower bud | 69 (36%) | 293 (63%) | 129 (77%) |
|  | Pollen | 121 (64%) | 172 (37%) | 38 (23%) |
| Correcting for degeneration of Y | | | | |
| Comparison of Differential expression | **Direction of overexpression** | **# Overexpressed (XY and X-only)** | **# Overexpressed (XY and X-only + ascertainment bias removed)** | |
| Leaf and pollen | Leaf | 193 (55%) | 141 (76%) | |
|  | Pollen | 158 (45%) | 44 (24%) | |
| Flower bud and pollen | Flower bud | 198 (55%) | 136 (74%) | |
|  | Pollen | 159 (44%) | 47 (26%) | |

Supplementary Table 2: Counts of XY genes with no allelic bias and Y and X biased allelic expression in *Rumex hastatulus and R. rothschildianus*.

| Sample | Tissue | # No allelic bias | # Y bias | # X bias |
| --- | --- | --- | --- | --- |
| *Rumex hastatulus* | | | | |
| 1 | Leaf | 236 | 28 | 23 |
| 2 | Leaf | 223 | 40 | 19 |
| 3 | Leaf | 165 | 38 | 38 |
| 4 | Flower bud | 283 | 20 | 22 |
| 5 | Pollen | 115 | 106 | 38 |
| 6 | Pollen | 120 | 77 | 22 |
| *Rumex rothschildianus* | | | | |
| 1 | Leaf | 61 | 57 | 156 |
| 2 | Leaf | 50 | 67 | 155 |
| 3 | Leaf | 81 | 67 | 137 |
| 4 | Flower bud | 52 | 45 | 178 |
| 5 | Flower bud | 58 | 48 | 172 |
| 6 | Pollen | 42 | 120 | 89 |
